# Metabolome of flue-cured tobacco is significantly affected by the presence of leaf stem

**DOI:** 10.1186/s12870-023-04093-2

**Published:** 2023-02-13

**Authors:** Yingxue Li, Fengfeng Liu, Shubin Sun, Yu Xiang, Xuebin Jiang, Jiewang He

**Affiliations:** 1 Technology Center, China Tobacco Hubei Industrial Co., LTD, Wuhan, 430040 Hubei China; 2 Xiangyang Cigarette Factory, China Tobacco Hubei Industrial Co., LTD, Xiangyang, 441000 Hubei China; 3 Enshi Cigarette Factory, China Tobacco Hubei Industrial Co., LTD, Enshi, 445000 Hubei China

**Keywords:** Tobacco, Postharvest treatment, Quality, Metabolites, Leaf

## Abstract

**Background:**

Leaves of tobacco (*Nicotiana tabacum* L.) are flue-cured to use as a key industrial supply in various parts of the world. The quality of tobacco leaves is dependent on chemical components and their proportions. Generally, the stem attached to tobacco leaf is detached before curing. However, the leaf stem remains green for an extended period of time (as compared to leaf) during flue-curing. Hence, it is expected to affect the quality of tobacco's final product.

**Results:**

To understand the impact of the green stem of leaf on the metabolome of flue-cured tobacco, we employed a broad targeted metabolomics approach. We selected two tobacco cultivars (Yun87 and K326) and cultivated them in five geographic locations in China. For flue-curing, leaves were harvested without a stem (L) or with an attached stem (SPL). After metabolome analysis, a total of 1027 metabolites were annotated in these samples. A variable number of metabolites were differentially accumulated between both types of leaves (depending on geographic location or cultivar) representing an influence of environment or genotype. Interestingly, only 68 metabolites were differentially accumulated between L and SPL samples irrespective of the cultivar or geographic location. These differentially accumulated metabolites belonged to major groups of primary and secondary metabolites. We have discussed the importance of identified metabolites in terms of carbon, nitrogen, and polyphenolic metabolism.

**Conclusion:**

The present research is the first comprehensive description of several metabolites in tobacco leaves related to the contribution of leaf stem. The current study opens novel prospects for investigating the potential of such metabolites in improving the quality of flue-cured tobacco.

**Supplementary Information:**

The online version contains supplementary material available at 10.1186/s12870-023-04093-2.

## Background

Tobacco (*Nicotiana tabacum* L.) is an industrial crop. It plays an essential economic and social role for many countries. Tobacco has a long history of cultivation around the world, which is used to prepare some special food products to be snuffed, chewed, sucked, or smoked [1]. In China, the overall yield of flue-cured tobacco leaves reached 2,994,500 tons in 2014 and represented > 90% of the total production, which has been the chief raw material in the cigarette industry. The quality of smoking food products relies on the composition of leaves. The processing method of tobacco leaves is also very crucial for the improvement of quality and preservation of flavor in these products. Curing is a fundamental practice in the primary processing of tobacco. It transforms agricultural products (tobacco leaves) into supplies for the tobacco industry. Leaf type, conditions, and curing technology significantly influence the flavor and quality of flue-cured tobacco products [2–4]. It is well documented that metabolites present in tobacco leaves are directly affected by climate as well as cultivated regions [2, 3]. On the other hand, cultivar factors also influence the quality or metabolic profile of tobacco leaves. Many flue-cured tobacco cultivars have been developed in China for different growing regions with significant environmental variations [5]. Different cultivars of tobacco have shown momentous variances in chemical components like alkaloids and aroma substances [6]. Fresh tobacco leaves (FTL) possess 80–85% moisture content and it remains only 16–18% after curing. During different drying stages, the curing conditions of FTL are optimized in accordance with the drying features [7] and thermal properties [8] of leaves. Furthermore, the moisture mobility and diffusion properties during drying are important aspects to consider when optimizing the curing conditions [9]. The influence of changes in these properties in tobacco leaves during drying and how they may participate in metabolic alterations related to tobacco quality are still unknown.

To estimate metabolite variations, plant metabolomics is considered a full scale and potent method that has been used in many plant-based fields like the assortment of biomarkers [10, 11], identification of gene function [12], GMO evaluation [13], atmospheric conditions [14, 15], growth stages [16] and documentation of variable metabolites and mechanisms among diverse cultivars [17, 18]. In the unconventional world of plant metabolomics, liquid chromatography-mass spectrometry (LC–MS), gas chromatography-mass spectrometry (GC–MS), nuclear magnetic resonance (NMR), and capillary electrophoresis–mass spectrometry (CE–MS) [19] are widely employed analytical methods. Multiple reaction monitoring (MRM)-based targeted metabolomics is a very refined and precise approach for measuring targeted metabolites with high throughput, high sensitivity, and broad coverage. This approach has been effectively utilized to identify many valuable metabolites in crop species such as *Oryza sativa* [20], *Sesamum radiatum* [21], and *Chrysanthemum morifolium* [22].

In the current study, two cultivars of tobacco (K326, Yun87) were cultivated in five geographical locations in China. Through the study of tobacco metabolic profiles, the differences in metabolites between the tobacco leaves cured with stems and the conventionally peeled/cured tobacco leaves were explored to explain the effect of stem curing on tobacco leaf quality. It is assumed that in the curing practice, the stem attached to tobacco leaves can contribute to a series of chemical reactions. We analyzed the metabolic profile of these flue-cured tobacco leaves in diverse geographical areas using broad target metabolome analysis and identified their differential metabolites previously never reported in tobacco. Moreover, metabolites independent of environment or cultivar effects were identified, which represented the specific contribution of leaf stems. Their involved metabolic pathways and the association of different metabolites were also analyzed.

## Materials and methods

### Experimental location and materials

**Fig. 1:**
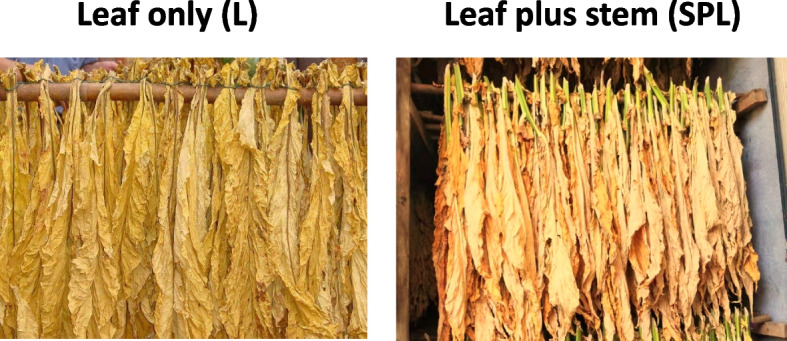
General representation of tobacco leaves used for metabolome analysis. The left panel represents the leaves normally used for flue-curing and the right panel represents the leaves along with green stems. For the analysis, only the six leaves present at the top of tobacco plant were used

Two cultivars of tobacco plants (K326 and Yun87) were cultivated at five geographic locations (Baokang, Xianfeng, Fangxian, Xuan'en, and Xingshan) in China in 2020 (Table 1). No permission is required to work on this species. Voucher specimens are available in the genebank herbarium Technology Center, China Tobacco Hubei Industrial Co., LTD. under the number: XTK96C77. Official identification of the plant material was conducted by Prof Jiewang He. Upper leaves (top 06 leaves) of these plants were harvested for analysis (Fig. 1). Both tobacco varieties K326 and Yun87 are high-quality flue-cured tobacco varieties with large planting areas in China, which have wide adaptability and good tobacco quality. Yun87 was bred by crossing Yunyan 2 as the female parent and K326 as the male parent. It is similar to K326 to a large extent, for example, the color of tobacco leaves is mostly golden or orange, the thickness of tobacco leaves is moderate, the oil content is more, the luster is strong, and the tissue is loose. Various chemical components are coordinated, the quality of the absorption is medium to high, the amount of aroma is sufficient, the concentration is medium, the odor is mixed, and the strength is moderate. The yield, average price, proportion of superior tobacco and output value of Yun87 are higher than those of the K326. Main meteorological conditions (longitude, latitude, mean daily temperature at different growth stages, transplanting-clumping stage rainfall, prosperous long-term rainfall, maturity rainfall, sunshine hours, and relative humidity) in the growing season (April–May, 2020) are presented in Supplementary table 1.

**Table 1:** Details of samples, geographic locations, and genotypes used in the current study

Sample groups	Sample ID	Geographic region	Variety	Name of sample	Type of Leaf
G1	L1V1L	Baokang (L1)	K326 (V1)	B2F-BK	Leaf only (L)
	L1V1SPL			BOH-BK	Leaf plus stem (SPL)
G2	L2V1L	Xianfeng (L2)	K326 (V1)	B2F-XF	Leaf only (L)
	L2V1SPL			BOH-XF	Leaf plus stem (SPL)
G3	L3V2L	Fangxian (L3)	Yun 87 (V2)	B2F-FX	Leaf only (L)
	L3V2SPL			BOH-FX	Leaf plus stem (SPL)
G4	L4V2L	Xuan'en (L4)	Yun 87 (V2)	B2F-XE	Leaf only (L)
	L4V2SPL			BOH-XE	Leaf plus stem (SPL)
G5	L5V2L	Xingshan (L5)	Yun 87 (V2)	B2F-XS	Leaf only (L)
	L5V2SPL			BOH-XS	Leaf plus stem (SPL)

(L1, L2, L3, L4, L5: Location, *V* Variety, *L* leaf only, *SPL* Stem plus leaf)

### Broad target metabolome analysis

Preparation of sample and extraction: Scientz-100F; A vacuum freeze-dryer was used to freeze-dry biological samples. A mixer mill (MM 400, Retsch) along with a zirconia bead was employed at 30 Hz for 1.5 min to crush freeze-dried samples. Lyophilized powder (100 mg) was dissolved in 1.2 ml of 70% methanol solution. The same sample was vortexed six times for 30 s at an interval of 30 min. After that, we refrigerated the samples at 4 °C overnight followed by centrifugation for 10 min at 12000 rpm. The extracts were filtrated (pore size 0.22 μm; SCAA-104, ANPEL, Shanghai, China) before UPLC-MS/MS analysis.

UPLC Conditions: an UPLC-ESI–MS/MS system (SHIMADZU Nexera X2 UPLC, Applied Biosystems QTRAP 4500) was employed for the analysis of samples. Following conditions were used in the analytical process, UPLC: column 1.8 µm, 2.1 mm*100 mm (Agilent SB-C18); solvent A, 0.1% formic acid in pure water, solvent B, 0.1% formic acid in acetonitrile and it was formed in the mobile phase. A gradient program (starting conditions of 95% A, and 5% B) was used to perform the sample’s measurements. A linear gradient was set in 9 min for 5% A and 95% B. Following that, a correction of 95% A and 5.0% B was performed in 1.10 min and kept for 2.9 min. The rate of flow was fixed at 0.35 ml/ min. The injection volume (4 μl) and the temperature (40° C) of the column oven were also attuned. The ESI-triple quadrupole-linear ion trap (QTRAP)-MS was coupled to the effluent as an alternative.

Triple quadrupole (QQQ) scans and ESI-QTRAP-MS/MS: LIT was obtained on a triple-quadrupole-linear ion trap mass spectrometer (QTRAP), AB4500 Q TRAP UPLC/MS/MS System equipped with an ESI Turbo Ion-Spray interface, running in both modes (negative and positive). A software package (Analyst 1.6.3, AB Sciex) was used for the control. Following parameters were employed for source operation of ESI: turbo spray, ion source; ion spray voltage of 5500 V (ion mode: positive)/ -4500 V (ion mode: negative); 550 °C source temperature; ion source gas I (GSI), curtain gas (CUR), gas II(GSII), were set at 50, 25.0 and 60 psi, respectively; the collision-activated dissociation (CAD) was high. LIT and QQQ modes, 100 and 10 mol/L polypropylene glycol solutions were used to turn the instrument and calibrate the mass. MRM experiments with nitrogen (collision gas set to the medium) were used for obtaining QQQ images. Individual MRM transitions were DP and CE optimized, with further DP and CE tuning. A set of specific MRM transitions was observed in accordance with the metabolites eluted in each phase. For metabolite identification by a widely targeted metabolomics approach, a self-compiled database called MetWare, (Wuhan MetWare Biotechnology Co., Ltd. (www.metware.cn)) was used. For this purpose, accurate m/z value of each precursor ion (Q1) was used [23]. This method has been previously described [24]. In brief, metabolites were identified by comparing the m/z values, the retention time (RT), and the fragmentation patterns with the standards in a self-compiled database (MetWare). Significantly changed metabolites (SCMs) were filtered according to |Log2 (fold change)|≥ 1, *p*-value < 0.05.

### Data processing and analysis

PCA: For performing an unsupervised PCA (principal component analysis), the statistics function was used in prcomp within R (www.r-project.org). Afore PCA, the data was unit variance scaled. HCA (Hierarchical Cluster Analysis) findings of metabolites in samples were shown as heatmaps. The cor function in R was used to calculate the pearson correlation coefficients (PCC) between samples. These were also demonstrated as heatmaps. The R ‘pheatmap’ package was used to do both PCC AND HCA. Signal intensities (normalized) of metabolites (at a scale of unit variance) are displayed as color spectra for HCA.

Assortment of differentially accumulated metabolites: Absolute log _2_ FC (fold change) ≥ 1 and VIP ≥ 1 were set to find distinct metabolites within groups. VIP data was retrieved from OPLS-DA results and the permutation plots. The R package (MetaboAnalystR) was used to generate score plots. The data was log converted (log _2_) and mean-centered before OPLS-DA. To avoid overfitting, the permutation test was performed using 200 permutations.

Analysis regarding KEGG annotation and enrichment: The identified metabolites were annotated using the KEGG database of compounds ( http://www.kegg.jp/kegg/compound/) and were mapped to the KEGG Pathway database ( http://www.kegg.jp/kegg/pathway.html). Metabolite sets enrichment analysis (MSEA) was used to map pathways of these metabolites and the hypergeometric test's p-values were used to determine their significance.

### Determination of conventional chemical compositions

The contents of total sugar, reducing sugar, total nitrogen, total alkaloids, chlorine, potassium, protein were determined by a continuous flow analyzer (Alliance-Futura), according to Tobacco Industry Standards YC/T159-2002, YC/T 161–2002, YC/T 468–2013, YC/T 217–2007, YC/T 162–2011, YC/T249–2008, respectively. Chlorogenic acid, neochlorogenic acid, scopoletin, rutin, caffeic acid, etc. were determined based on Chinese specifications for High-performance Liquid Chromatography for determining chlorogenic acid, scopoletin, and rutin in Tobacco and Tobacco products (YC/ T202–2006).

## Results

### Metabolome analysis

**Fig. 2:**
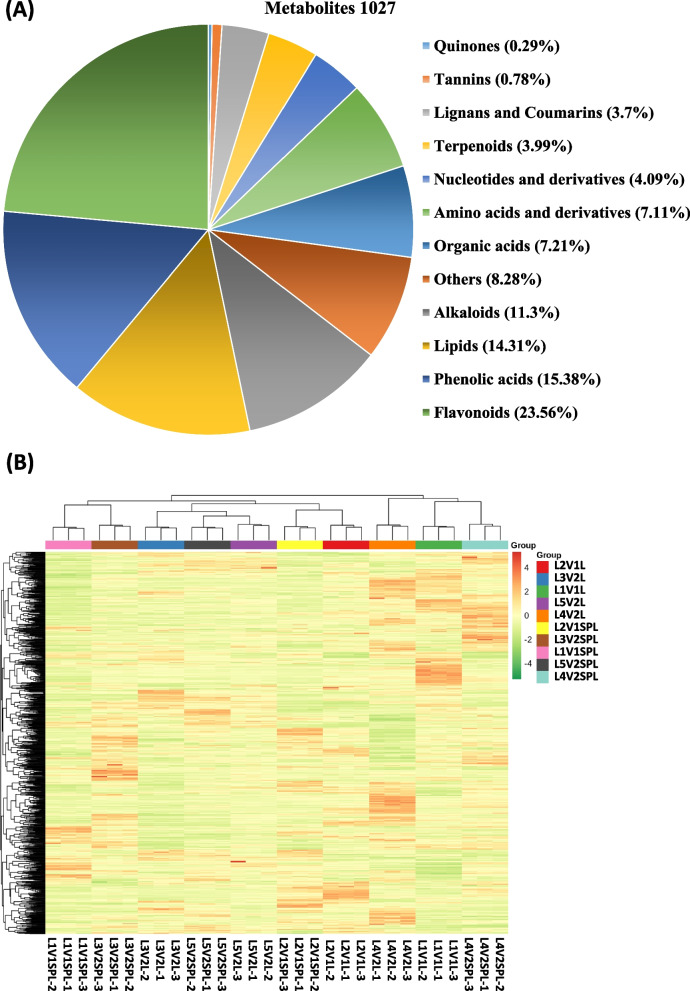
A general description of identified metabolites. **A** Classification as well as the composition of the 1027 metabolites of tobacco leaves. **B** Heatmap regarding hierarchical clustering analysis (HCA) of metabolites of all samples. Location of cultivation (L1, L2, L3, L4, L5), Genotype (V1, V2), only leaf (L), stem plus leaf (SPL)

The total ion chromatogram (TIC) of a mixture of all the investigated samples (the quality control, QC), as well as chemicals’ multi-peak detection plot in the MRM mode of the same sample, is shown in Supplementary Figure S1. Different components of the samples are represented by different colored peaks. As shown in Fig. 2, the current study resulted in the identification of 1027 metabolites (Supplementary table 2). These metabolites are divided into 12 classes, including 03 Quinones, 08 Tannins, 38 Lignans and Coumarins, 41 Terpenoids, 42 Nucleotides/derivatives, 45 Saccharides and Alcohols, 74 Organic acids, 72 Amino acids/derivatives, 40 others, 116 Alkaloids, 147 Lipids, 158 phenolic acids, and 242 Flavonoids. Among these compounds, the largest group was Flavonoids, which accounted for 23.56% of the total metabolite composition in terms of relative content. Furthermore, Alkaloids, Lipids, and Phenolic acids also represented major groups with 11.29%, 14.31%, and 15.38%, respectively (Fig. 2).

**Fig. 3:**
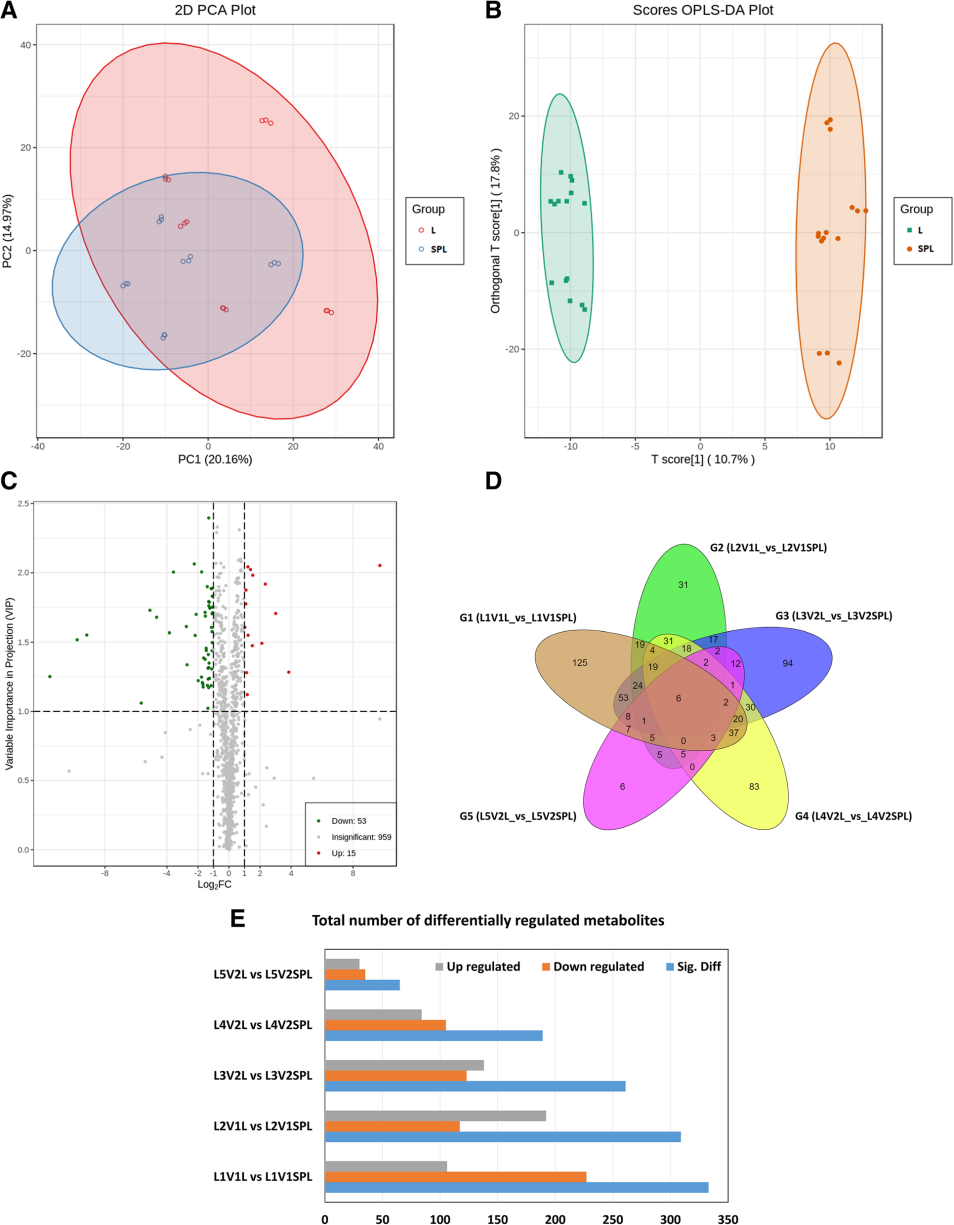
Analysis of differential metabolite in all samples. **A** Principal component analysis to visualize the sample distributions, **B** OPLS-DA generated score plots of the differential metabolites; **C** The volcano plot representing differential metabolites among different samples; **D** the Venn diagram showing differential metabolites in different samples, **E** Total number of differentially accumulated metabolites in different samples. Location of cultivation (L1, L2, L3, L4, L5), Genotype (V1, V2), only leaf (L), stem plus leaf (SPL). OPLS-DA, orthogonal partial least squares discriminant analysis

HCA evaluated the metabolite accumulation pattern in different treatment groups. As shown in Fig. 2B, the Heatmap of 1027 metabolites is clustered as a heatmap (based on Euclidean distance arithmetic). Identified metabolites in both sample types (leaves without or with stems) at five geographical locations in two genotypes were grouped into distinct clusters according to the dendrogram. The brighter the color, the higher the concentration of a particular metabolite in the respective sample. The HCA’s heatmap showed larger differences in abundance among samples. For location 5 (see Table 1) metabolites from both types of samples were clustered together (L5V2L and L5V2SPL). Similarly, metabolite samples from location 2 were also clustered together. However, samples from locations 1, 3, and 4 were not present in the same clusters (Fig. 2B). Genotype 1 (V1, please see Table 1) was cultivated at two locations (L1 and L2). Metabolites from both locations for this genotype were present in different clusters. Genotype 2 (V2) was cultivated at three different locations (L3, L4, and L5). Metabolites for genotype 2 shared a cluster for L3 and L5, while metabolites from L4 were present in a separate group. In other words, both genetic and geography-dependent variations were observed for metabolites. However, distinct metabolite profiles were observed for both types of samples independent of geographic locations (Fig. 2B). Principal component analysis (PCA) revealed that the first and second principal components accounted for 20.16 and 14.97% of the total variance, respectively (Fig. 3A). The analysis of the metabolite profiles by PCA revealed a clear separation of all treated samples. Figure 3B is showing the OPLS-DA scatter scores regarding pairwise comparison groups. It shows that metabolites from both types of samples were significantly different regardless of location and genotype. Furthermore, high-test values for Q2 and R2Y demonstrated that this model was highly significant without overfitting (Supplementary Figure S2).

To study the expression levels of metabolites in both types of tobacco samples (Leaf only vs stem plus leaf), the volcano plot was again generated among all 1027 identified metabolites based on fold-change and VIP values were coupled with them. Distinct differential metabolites were chosen based on the criteria, which included a fold change score of ≥ 2 or ≤ 0.5 with a VIP ≥ 1. Figure 3C shows the results of screening. The color of the scattered dots on the volcano plot shows the results of final screening. Significantly up-accumulated (UA) metabolites are represented in red, and significantly down-accumulated (DA) metabolites are represented in green. Insignificantly different metabolites are represented as gray. Figure 3C shows that 68 metabolites (15 UA and 53 DA) were differentially accumulated.

Although our study did not precisely target nicotine, we compared nicotine levels using untargeted data as well as its derivatives profiles among these samples. Following metabolites related to Nicotine were identified: Nicotine, Nicotine-N-glucuronide, 6-Hydroxynicotine, Nornicotine, Nicotine-N-glucoside, Cotinine, Cotinine-glucoside, and 3'-Hydroxycotinine (Supplementary table 2). However, none of these metabolites were differentially accumulated in L vs SPL samples in overall or individual pairwise comparisons (Table 2). In conclusion, these findings demonstrate that the nicotine levels are trending independent of the presence or absence of leaf stem. Similar findings indicated the absence of correlation in nicotine contents, genotypes, and farming locations [25].

**Table 2:** List of significantly different metabolites up/down-accumulated in Leaf plus stem (LPS) samples as compared to leaf only (L) samples

Index	Compounds	Class I	Class II	Fold_Change	Accumulation
pme3882	2'-Deoxyuridine	Nucleotides /derivatives	Nucleotides /derivatives	846.82	up
Lmhp009464	LysoPE 17:1(2n isomer)	Lipids	LPE	14.46	up
pmc0066	2'-Deoxyinosine-5'-monophosphate	Nucleotides /derivatives	Nucleotides /derivatives	8.06	up
pme0264	Thymidine	Nucleotides /derivatives	Nucleotides /derivatives	5.07	up
Lmhp008337	LysoPE 14:0(2n isomer)	Lipids	LPE	4.35	up
Lmhp007598	LysoPC 19:2(2n isomer)	Lipids	LPC	2.89	up
pme1184	2'-Deoxyguanosine	Nucleotides /derivatives	Nucleotides /derivatives	2.83	up
pmp000117	3,5,6,7,8,3',4'-Heptamethoxyflavone	Flavonoids	Flavonols	2.62	up
pmb2260	LysoPC 15:1	Lipids	LPC	2.35	up
Lmhp007836	LysoPE 16:3	Lipids	LPE	2.33	up
Smpn009230	2α,3α,23-trihydroxyolean-12-en-28-oic acid	Terpenoids	Triterpene	2.26	up
pmn001587	Asperulosidic acid	Terpenoids	Monoterpenoids	2.19	up
Lmhp010515	LysoPC 17:0(2n isomer)	Lipids	LPC	2.14	up
Lmhp007840	LysoPC 19:2	Lipids	LPC	2.13	up
Lmhp008718	LysoPC 17:2	Lipids	LPC	2.03	up
Lmmn001643	2-Hydroxycinnamic acid	Organic acids	Organic acids	0.48	down
pme3033	N,N-Dimethylglycine	Amino acids/derivatives	Amino acids/derivatives	0.48	down
Zmzn000113	L-threo-3-Methylaspartate	Amino acids/derivatives	Amino acids/derivatives	0.47	down
Zmyn004449	9-Hydroxy-12-oxo-10(E),15(Z)-octadecadienoic acid	Others	Others	0.47	down
pmb0069	Benzamide	Phenolic acids	Phenolic acids	0.47	down
pmb3066	5-O-p-Coumaroylshikimic acid O-glucoside	Phenolic acids	Phenolic acids	0.47	down
mws0254	L-Histidine	Amino acids/derivatives	Amino acids/derivatives	0.47	down
HJN104	Dihydromyricetin-3-O-glucoside	Flavonoids	Dihydroflavonol	0.47	down
Lmgn000160	3-Ureidopropionic acid	Organic acids	Organic acids	0.46	down
mws0491	Phenethylamine	Alkaloids	Alkaloids	0.46	down
Lmbn002648	α-Hydroxycinnamic acid	Phenolic acids	Phenolic acids	0.45	down
mws0489	Benzoylformic acid	Organic acids	Organic acids	0.45	down
pme3011	γ-Aminobutyric acid	Organic acids	Organic acids	0.43	down
Zmgn001448	2-Propylmalic acid*	Organic acids	Organic acids	0.43	down
pme3217	Isoliquiritigenin	Flavonoids	Chalcones	0.42	down
pmb3101	2-Isopropylmalic Acid*	Organic acids	Organic acids	0.41	down
mws0206	2-Hydroxybutyric Acid*	Organic acids	Organic acids	0.41	down
Lmbn001754	3-Isopropylmalic Acid*	Organic acids	Organic acids	0.41	down
pme2693	N-Acetylputrescine	Alkaloids	Phenolamine	0.41	down
Lmrj002244	Cyclo(Pro-Pro)	Amino acids/derivatives	Amino acids/derivatives	0.40	down
pme0278	2,6-Diaminopimelic acid	Amino acids/derivatives	Amino acids/derivatives	0.40	down
Wmzn000227	2,2-Dimethylsuccinic acid	Organic acids	Organic acids	0.40	down
mws1105	Gramine	Alkaloids	Alkaloids	0.40	down
pme3968	7-Methylguanine	Nucleotides /derivatives	Nucleotides /derivatives	0.39	down
pmb0501	Agmatine	Alkaloids	Phenolamine	0.39	down
pmp001189	(5–8)-Hydroxy-1-(hydroxyldimethoxyphenyl)-N2,N3-bis(4-hydroxyphenethyl)-(5–8)-dimethoxy-1,2-dihydronaphthalene-2,3-dicarboxamide	Alkaloids	Alkaloids	0.39	down
pmb0776	N-Feruloyltryptamine	Alkaloids	Phenolamine	0.38	down
Zmgn002106	N-Acetyl-L-phenylalanine	Amino acids/derivatives	Amino acids/derivatives	0.38	down
Zmjp000182	N-Monomethyl-L-arginine	Amino acids/derivatives	Amino acids/derivatives	0.36	down
mws0346	3-(3-Hydroxyphenyl)-propionic acid	Organic acids	Organic acids	0.36	down
pme0193	L-Glutamine	Amino acids/derivatives	Amino acids/derivatives	0.35	down
mws0574	2-Hydroxyisobutyric acid*	Organic acids	Organic acids	0.34	down
pme0026	L-Lysine	Amino acids/derivatives	Amino acids/derivatives	0.34	down
mws0260	L-Arginine	Amino acids/derivatives	Amino acids/derivatives	0.32	down
Lmlp003161	N-Feruloylputrescine	Alkaloids	Phenolamine	0.32	down
pme3388	Homoarginine	Amino acids/derivatives	Amino acids/derivatives	0.31	down
pmb0962	L-Lysine-Butanoic acid	Amino acids/derivatives	Amino acids/derivatives	0.30	down
mws0715	Phenylacetyl-L-glutamine	Amino acids/derivatives	Amino acids/derivatives	0.29	down
pme0266	Sebacate	Organic acids	Organic acids	0.29	down
Lmmp002013	Dihydroferuloylputrescine	Alkaloids	Phenolamine	0.25	down
pme2292	Putrescine	Alkaloids	Phenolamine	0.23	down
mws0001	L-Asparagine	Amino acids/derivatives	Amino acids/derivatives	0.22	down
Lmhp001732	L-Prolyl-L-Phenylalanine	Amino acids/derivatives	Amino acids/derivatives	0.21	down
pmb0490	p-Coumaroylputrescine	Alkaloids	Phenolamine	0.15	down
pmb0323	N-Caffeoylputrescine	Alkaloids	Phenolamine	0.15	down
Lmmp002914	Bis(Dihydrocaffeoyl)spermidine	Alkaloids	Phenolamine	0.08	down
pmn001726	N1-Caffeoyl-N3-dihydrocaffeoyl spermidine	Alkaloids	Phenolamine	0.07	down
Lmmp003167	Bis(Caffeoyl)Spermidine	Alkaloids	Phenolamine	0.04	down
Lmmp003112	Caffeoyl-dihydrocaffeoyl spermidine	Alkaloids	Phenolamine	0.03	down
mws1562	Catalpol	Terpenoids	Terpene	0.02	down
Lmhn003223	Cinnamoyltartaric acid	Phenolic acids	Phenolic acids	0.00	down
MWSslk144	p-Coumaric acid ethyl ester	Organic acids	Organic acids	0.00	down
mws0425	Citraconic acid	Organic acids	Organic acids	0.00	down

These differential metabolites were further classified as well as compared. The classification of these differentially accumulated metabolites was performed into 09 classes, principally alkaloids, amino acids/derivatives, organic acids, lipids, nucleotides/derivatives, phenolic acids, flavonoids, terpenoids, and others (Table 2). It can be clearly found that Organic acids (including Citraconic acid, and p-Coumaric acid ethyl ester, Sebacate), Phenolic acids (Cinnamoyltartaric acid), Terpenoids (Catalpol), Alkaloids (including Caffeoyl-dihydrocaffeoyl spermidine, Bis(Caffeoyl)Spermidine, N1-Caffeoyl-N3-dihydrocaffeoyl spermidine, Bis(Dihydrocaffeoyl)spermidine, N-Caffeoylputrescine, p-Coumaroylputrescine, Putrescine), Amino acids and derivatives (L-Prolyl-L-Phenylalanine, L-Asparagine, Phenylacetyl-L-glutamine, L-Lysine-Butanoic Acid, Homoarginine, L-Arginine, L-Lysine, L-Glutamine) were significantly DR, while Nucleotides and derivatives (including 2'-Deoxyuridine, 2'-Deoxyinosine-5'-monophosphate, Thymidine, 2'-Deoxyguanosine), Flavonoids ( 3,5,6,7,8,3',4'-Heptamethoxyflavone), Terpenoids ( 2α,3α,23-trihydroxyolean-12-en-28-oic acid, Asperulosidic acid) and lipids ( LysoPE 17:1(2n isomer), LysoPE 14:0(2n isomer), LysoPC 19:2(2n isomer), LysoPC 15:1, LysoPE 16:3, LysoPC 17:0(2n isomer), LysoPC 19:2, LysoPC 17:2) were significantly UR. It is interesting to notice that Nucleotides/derivatives and lipids represented the majority of up-accumulated metabolites in samples representing stem plus leaf samples (Table 2).

A Venn diagram was generated to differentiate the common and specific metabolites of pairwise comparisons. Figure 3D shows that unique, as well as common metabolites, exist among various comparison groups. A variable number of metabolites were differentially accumulated between leaf only and stem plus leaf groups of two genotypes and five locations (Supplementary table 3, Fig. 3E). A total of 125, 31, 94, 83, and 6 exclusive metabolites (*p* < 0.05) were observed in G1, G2, G3, G4, and G5 respectively. These findings added to the evidence that the presence or absence of leaf stem was significantly important for the metabolites conversion during leaf curing.

**Fig. 4:**
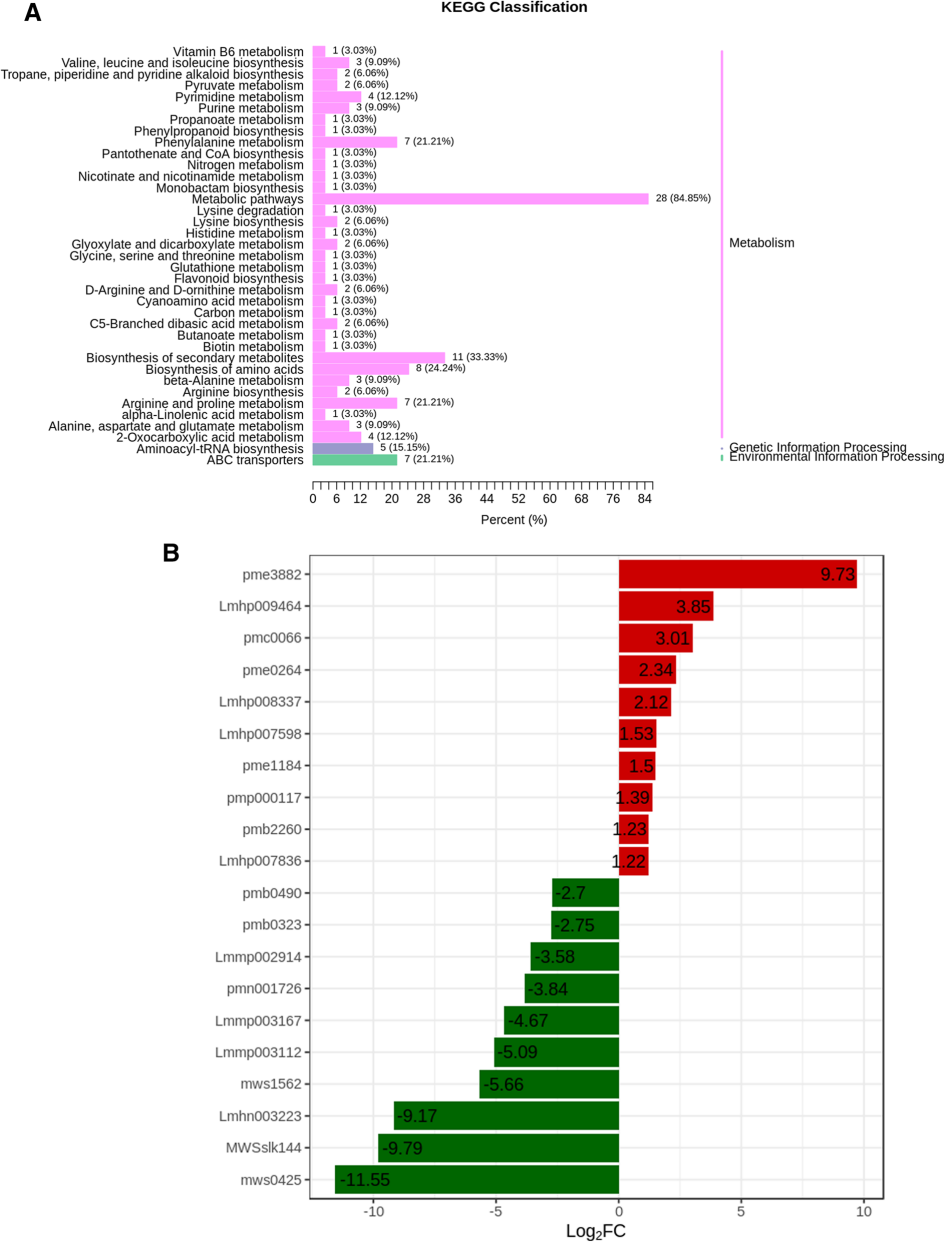
Analysis of metabolic enrichment pathway in two comparative groups. **A** KEGG classification, **B** Enriched compounds with the highest fold change

Differential metabolites in samples with *p* < 0.05 were mapped to the KEGG database (Fig. 4). Figure 4A shows that the enrichment of pathways was revealed as a result of metabolic pathways, biosynthesis of secondary metabolites as well as amino acids, Phenylalanine metabolism, and Proline and Arginine metabolism. The occurrence of differentially exclusive metabolites could be explained by changes in metabolic pathways. Among the most differentially up-accumulated (Table 2) metabolites were Lipids (8 metabolites), Nucleotides/derivatives (4 metabolites), Terpenoids (2 metabolites), and Flavonoids (1 metabolite). Similarly, 15Alkaloids, 15 Amino acids as well as their derivatives, 14 Organic acids, 04 Phenolic acids, 02 Flavonoids, 01 Nucleotide/derivatives, 01 Terpenoid, and 01 Others were down-accumulated. Interestingly, Lipids represented the highest up-accumulated metabolites. However, amino acids/derivatives, phenolic acids, organic acids, alkaloids, and nucleic acids/derivatives were mostly down-accumulated in SPL samples.

### Physiological and biochemical properties

**Fig. 5:**
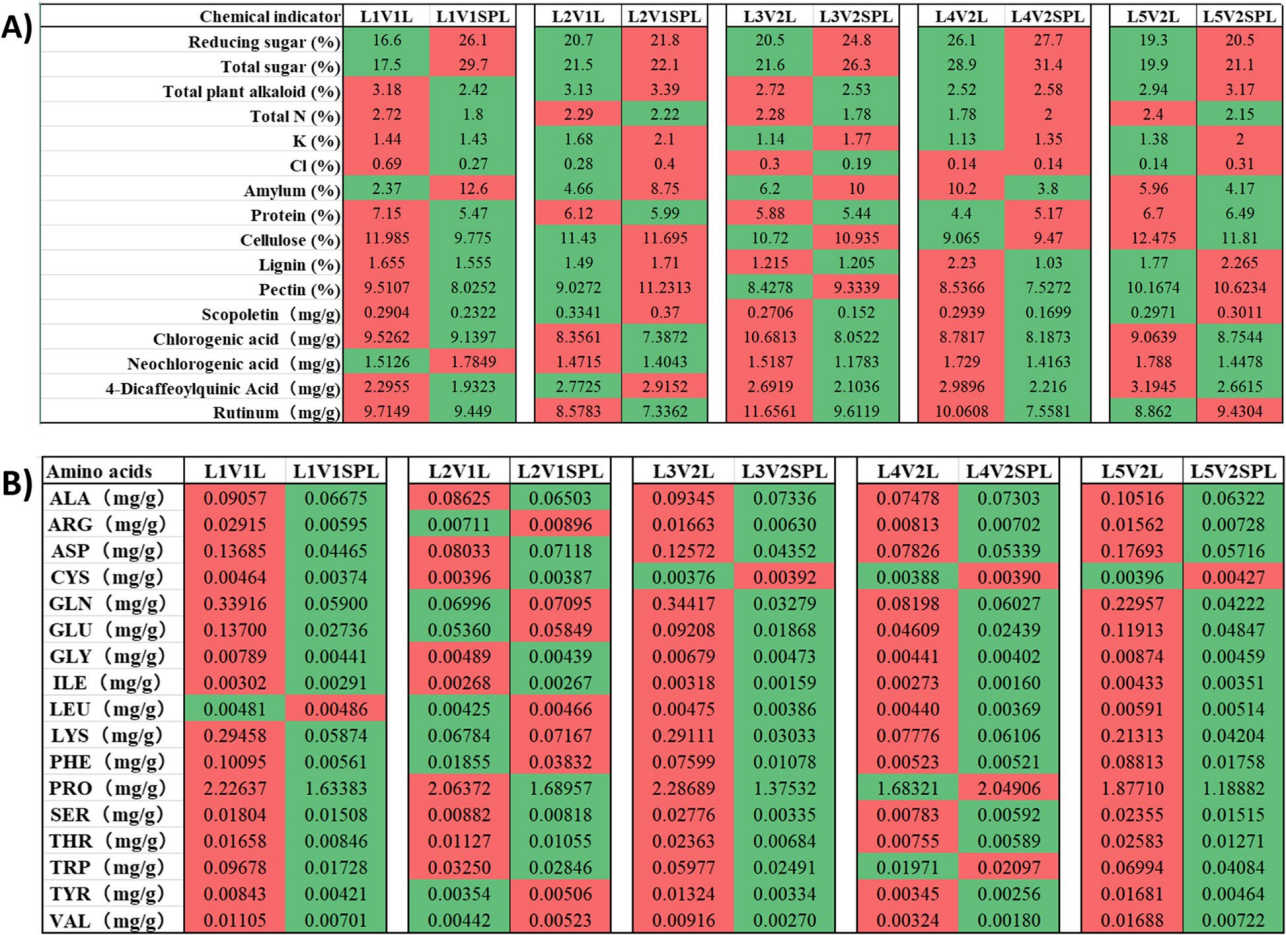
Physiological and biochemical properties. **A** Chemical indices and Polyphenol substances composition of tobacco leaves, **B** Amino acid composition of tobacco leaves. Red represents higher content and green represents lower content. L1, L2, L3, L4, and L5 represent five geographic locations. V1 and V2 represent two varieties of tobacco. L and SPL represent Leaf only and Stem plus leaf, respectively. Three-letter codes represent amino acids

A visible trend of variation was observed for studied physiological and biochemical properties among all the samples studied (Fig. 5). Higher amounts of reducing sugars and total sugars were present in SPL samples among all groups. Total nitrogen and total potassium contents were mostly lower in SPL and L samples, respectively (4 out of 5 groups). Total protein contents were mostly lower in SPL samples as compared to the other samples (Fig. 5A). However, variations in Cl, total Alkaloids, Amylum, cellulose, lignin, and pectin seemed affected by location or genotype. The quantitative variations in polyphenol substances were either inconclusive (scopoletin) or decreased in all or at least four sample groups of SPL (Fig. 5). Amino acid profiles of all samples were estimated to understand nitrogen metabolism. It was observed that all amino acids (except LEU and CYS) were present in fewer quantities in SPL samples as compared to L (Fig. 5B).

## Discussion

Through the study of tobacco metabolic profiles, the differences in metabolites between the upper tobacco leaves cured without stems (L) and leaves cured with stems (SPL) were explored to explain the effect of stem curing on tobacco leaf quality of two cultivars (K326 and Yun87) at five different locations in China. The quality of tobacco leaves is directly related to the chemical substances and their portion present in them, which in turn determines the smoking grade of cigarettes [26]. The current study reported ~ 1027 metabolites in flue-cured upper tobacco leaves related to 12 different classes. Individual metabolite classes have also been quantified in flue-cured tobacco leaves in previous research [3, 27–29]. Most of these studies reported a limited number of metabolites related to a particular class or group of compounds.

The growth conditions and curing conditions for L and SPL-related plants are the same and only the harvesting method is different. The metabolic profiles of the tobacco cultivars K326 and Yun87 were considered similar to each other as compared to other cultivars [30]. In the current study, quantitative variations of various metabolites were identified that were specific to each genotype or location of cultivation (Fig. 3C). However, on a global scale (among five locations and two cultivars) only 68 metabolites were differentially accumulated. It potentially corresponds to the presence or absence of the leaf stem (Table 2). According to KEGG classification, the majority of differentially accumulated pathways represented metabolic pathways, biosynthesis of secondary metabolites, and metabolism of amino acids (Fig. 4). A recent study of flue-curing with or without leaf stems has reported a significant effect of stem on metabolome [31]. Compared with stem( +), 34 metabolites were down-accumulated and 126 were up-accumulated from that of stem(-). Moreover, Indole-3-acetic acid (IAA) was the second most differential metabolite, which was abundant in stem(-) but did not exist in stem( +). The chemical composition of flue-cured tobacco leaves is very complex, in which thousands of chemical compounds have been identified [32, 33]. In tobacco leaves, carbon metabolism is the most basic kind of metabolism that occurs during the flue-curing [34]. A study conducted by Gong and Yamaguchi [35–37] showed the breakdown of starch into water-soluble sugars during flue-curing which are then accumulated into the leaves. In this way, soluble sugars are considered the highest accumulated content in leaves that swiftly degrade at the yellowing stage of tobacco leaves. In the current study, an overall higher percentage of Reducing sugars and Total sugars were observed in SPL samples (Fig. 5A). On the other hand, cellulose, lignin, and pectin were mostly decreased (all or at least one) in SPL samples. Total sugar and reducing sugars, on the other hand, have a substantial negative association with indices like coordination and taste [38]. Similarly, when the effect of oven-drying and flue-curing was observed in tobacco leaves, it was observed that compared with oven-drying, starch content significantly decreased while saccharides (including total sugar, glucose, fructose, maltose, and sucrose) increased in flue-cured tobacco leaves. There was only a numerical change in reducing-sugar content between the two drying methods [28]. A possible reason for this is that the rate of metabolic activities was different (potentially in the initial phase) in SPL as compared to L samples. It is speculated that Maillard reactions transform sugars into aroma components during the middle and late stages of flue-curing [28, 39].

Many changes in proteins and amino acid structure have been reported in tobacco leaves during processing (curing and storage). Decarboxylation and deamination of amino acids form dicarbonyl compounds, which react with the amino group (–NH_2_) of amino acids by the loss of CO_2_. This degradation process continues and produces pyrazine compounds that affect the taste as well as the perception of smoking cigarettes. The –NH_2_, primary and secondary amines, and ammonia form adducts with carbonyl compounds. During pyrolysis, it yields chemicals with non-enzymatic browning processes that are major flavor ingredients in smoke. The percentage of total N, proteins, and amino acids/ derivatives decreased in SPL samples as compared to L samples (Fig. 5, Table 2). It is possible that proteins were degraded during the flue-curing of tobacco leaves and it also promoted the loss of nitrogen [28].

In this study, the percentage of K contents was mostly higher in SPL samples and Cl contents were inconclusive. The smoking quality of flue-cured tobacco is directly influenced by the K and Cl content. The high K content helps to improve hygroscopicity, combustibility as well as characteristic identity and color of tobacco leaves [40]. On the other hand, Cl content is responsible for a better yield of leaves as well as for improved water content, elasticity, and storage features; however, too much Cl can obstruct sugar metabolism, allowing too much starch to accumulate, causing thickening and embrittling of the leaves [41].

Polyphenols are the compounds that boost the quality of tobacco leaves due to the readily oxidizing property of their yield red-brown and dark brown substances [33], which are responsible for the darkening of cigar leaves. Chlorogenic acid, neochlorogenic acid, 4-dicaffeoylquinic acid, rutinum, and scopoletin are all major phenolic compounds found in tobacco leaves, accounting for more than 80% of the phenolic content. These chemicals have a significant impact on the quality of tobacco leaves [42], thereby determining their contents in tobacco leaves is critical. In the SPL leaves, rutinum, and scopoletin contents were mostly decreased and the chlorogenic acid was decreased in all the SPL samples (Fig. 5). Such a decline in chlorogenic acid is attributable to the fact that chlorogenic acid serves as the main precursor of brown-colored products [43]. Phenolic hydroxyl groups, one of the common properties of phenolic substances, have strong reducing properties. According to Chen et al. [44], polyphenol oxidase (PPO) species are generated by membrane lipid peroxidation and loss of hydroxyl group by the oxidation of polyphenols, which in turn, produce quinones. Afterward, a long chain of reactions yields brown compounds that directly imparted color properties to tobacco leaves. Since most of the differentially down-accumulated metabolites belong to phenolic acids (Table 2), the current study proposes the existence of a similar mechanism [44–46].

Nucleotides, their derivatives, and lipids represented the majority of differentially accumulated metabolites between SPL and L samples. It is well-established that lipids are related to taste and aroma [47–50]. However, it is yet unknown how nucleotides/derivatives are involved in this process. Further investigation in this context would provide valuable information that could eventually be used to control the taste of a cigarette. It was reported that PI, PS, PE, and PC are not degraded during flue-curing. Dunkle et al. [51] discussed lipids in tobacco leaves in terms of curing type-specific lipids. In current study, lipids from two classes (LPC and LPE) were upaccumulated in SPL samples (Table 2). These compounds can be used to differentiate lipid-dependent quality-related traits in tobacco leaves.

## Conclusion

The present study reported the metabolic profile of two types of tobacco leaf samples (with or without leaf stem) from two cultivars cultivated in five geographic locations. Apart from the effects of genotype and cultivation area, we observed distinct contributions of leaf stem in metabolomic profiles. Hence, this information can be used in further investigations to understand the effect of leaf stems on the flue-curing process of tobacco leaves. Moreover, the comprehensive metabolite catalog described in the study can supplement the prevailing collection of tobacco metabolomics data.

## Supplementary Information

**Additional file 1:** **Supplementary Figure S1. **Multiple reaction monitoring (MRM) detection of multimodal maps (A for positive mode and B for negative mode).

**Additional file 2:** **Supplementary Figure S2.** Permutation test for OPLS-DA model for pairwise comparison of metabolic profiles of tobacco leaves.

**Additional file 3:** **Supplementary table 1:** Main meteorological conditions in the growing season of tobacco leaves.

**Additional file 4:** **Supplementary table 2. **Complete metabolome of SPL and L tobacco leaves.

**Additional file 5:** **Supplementary table 3. **Differentially regulated metabolome of each comparison group.

## Data Availability

All data used in this paper are available within the text and the supplementary files.
