# Supplementary figures and images for: Metabolome of flue-cured tobacco is significantly affected by the presence of leaf stem
Source: BMC Plant Biol. 2023 Feb 13;23:89. doi: 10.1186/s12870-023-04093-2 (PMC9926566; doi:10.1186/s12870-023-04093-2)

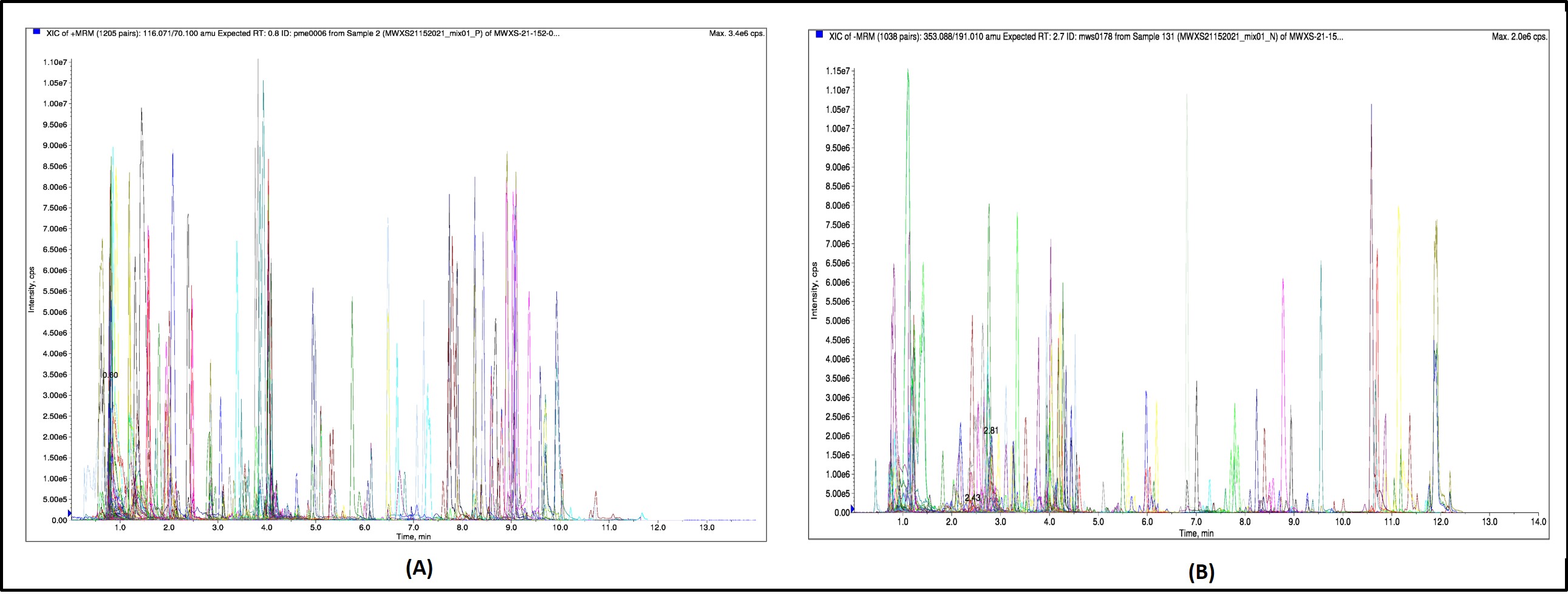

Supplement: Supplementary file 1 — Additional file 1: Supplementary Figure S1. Multiple reaction monitoring (MRM) detection of multimodal maps (A for positive mode and B for negative mode). [file 12870_2023_4093_MOESM1_ESM.jpg]

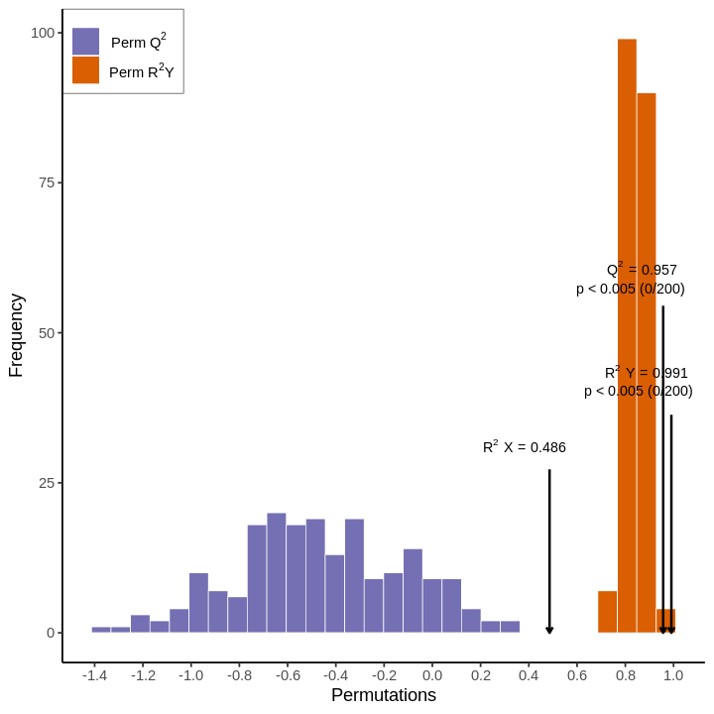

Supplement: Supplementary file 2 — Additional file 2: Supplementary Figure S2. Permutation test for OPLS-DA model for pairwise comparison of metabolic profiles of tobacco leaves. [file 12870_2023_4093_MOESM2_ESM.jpg]
